# Supplementary material for: Content-rich biological network constructed by mining PubMed abstracts
Source: BMC Bioinformatics. 2004 Oct 8;5:147. doi: 10.1186/1471-2105-5-147 (PMC528731; doi:10.1186/1471-2105-5-147)
Supplement: Additional File 2 — The original results of the above study (non-essential files are deleted to keep the file size under the limit set by BMC bioinformatics). [file 1471-2105-5-147-S2.bz2 › chilibotAdditionalFile2/dip20/15ID11821039E61/html/CARB_CARA.html]

 


 **CARB** and **CARA** 
  
Found 27 abstracts in PubMed, retrieved 20.  
 

 What does Google say? 
 PDF only 
| .edu only 

---

**Interactive relationship** (e.g. stimulation, inhibition, etc)

**Neutral relationship**- ...  **carA**  is linked to  **carB** .  Ref: 7968516 Mol Microbiol, 1993
- Sequencing has shown that CPS A  [ **CARA** ]  is encoded by  **carA**  GLN and  **carB**  SYN.  Ref: 10852872 J Bacteriol, 2000
- **CarA**  represses transcription from the  **carB**  promoter P B in the dark, and CarS counteracts this on illumination.  Ref: 11748235 J Biol Chem, 2002
- The cloned R. meliloti  **carA**  and  **carB**  genes were unable to complement E. coli  **carA**  or  **carB**  mutants alone or in combination.  Ref: 2846756 J Gen Microbiol, 1988
- A cis dominant mutation in the gene  **carA**  causes constitutive expression of the light inducible gene  **carB** , which is linked to  **carA** .  Ref: 2123519 Mol Gen Genet, 1990
- Among Bacteria the  **carA**  and  **carB**  genes encoding the small  **CarA**  and large  **CarB**  subunits of carbamoylphosphate synthase CPS have been lost in certain symbionts Haemophylus influenzae and in most obligate intracellular parasites Chlamydiae, Spirochaetes, Mycoplasmatales, Rickettsiae having genome sizes in the 0.7 to 1.1 Mb range.  Ref: 12107592 J Mol Evol, 2002
- **CarA**  hereafter named carbapenam synthetase has been proposed to catalyze formation of 3S, 5S carbapenam 3 carboxylic acid from 2S, 5S 5 carboxymethyl proline based on characterization of the products of fermentation experiments in Escherichia coli cells transformed with pET24a  **carB**  and pET24a carAB, and on sequence homology to beta lactam synthetase, an enzyme that catalyzes formation of a monocyclic beta lactam ring with concomitant ATP hydrolysis.  Ref: 12820893 Biochemistry, 2003
- Since a mutation in  **carB**  blocks accumulation of phytoene, we propose that  **carB** , and probably other linked genes also controlled by  **carA** , code for enzymes involved in the synthesis of phytoene.  Ref: 2123519 Mol Gen Genet, 1990

**Non-interactive relationship** (e.g. studied together, co-existance, homology, etc.)

- To determine whether the intervening sequence observed in N. gonorrhoeae CH811 was not unusual, the sequence between  **carA**  and  **carB**  was amplified by PCR from 30 isolates of N. gonorrhoeae.  Ref: 7773412 Microbiology, 1995
- We have determined the complete DNA sequence of the  **carA**  and  **carB**  genes of Neisseria gonorrhoeae strain CH811.  Ref: 7773412 Microbiology, 1995
- Identification of greA encoding a transcriptional elongation factor as a member of the  **carA**  orf  **carB**  greA operon in Pseudomonas aeruginosa PAO1.  Ref: 9139926 J Bacteriol, 1997
- The Erwinia carotorova  **carA** ,  **carB** , and carC gene products are essential for the biosynthesis of 5R carbapen 2 em 3 carboxylic acid, the simplest carbapenem beta lactam antibiotic.  Ref: 12820893 Biochemistry, 2003
- DNA sequence analysis reveals a proteobacterial architecture, namely closely linked  **carA**  and  **carB**  coding domains separated by a short intergenic region, and transcribed as a polycistronic mRNA.  Ref: 10675618 Gene, 2000
- S1 nuclease experiments showed that level of the greA transcript is elevated approximately 10 fold under conditions of pyrimidine limitation, consistent with the conclusion that transcription is initiated from the previously identified pyrimidine sensitive promoter upstream of the  **carA**  orf  **carB**  greA operon.  Ref: 9139926 J Bacteriol, 1997
- Whereas  **carA**  and  **carB**  are separated by a short noncoding intercistronic region, the homologous sequences of the CAD gene encode an amino acid bridge.  Ref: 1518089 J Mol Evol, 1992
- S1 nuclease mapping experiments with RNA extracted under a variety of growth conditions, as well as experiments using different lacZ fusions, indicate that the  **carA**  ORF  **carB**  operon of P. aeruginosa is transcribed from a single promoter.  Ref: 8169201 J Bacteriol, 1994
- Five possibly six genes in the  **carA**   **carB**  operon code for enzymes acting on early or late steps of the pathway for carotenoid synthesis.  Ref: 7588751 Eur J Biochem, 1995
- Alignment of the enzyme sequence derived from URA2 with sequences from Escherichia coli  **carA**   **carB**  and yeast arginine specific CP A1 CP A2 indicates that monofunctional and bifunctional carbamoyl phosphate synthetases are probably homologous.  Ref: 3039294 Mol Gen Genet, 1987
- Group 3 taxa the crenarchaea Pyrobaculum aerophilum, Sulfolobus solfataricus, and Sulfolobus tokodaii and the euryarchaeon Pyrococcus furiosus harbor CPS genes whose encoded proteins appear to be archaeal consistent with an archaeal origin, the  **CarA**  and  **CarB**  sequences in this group possess both unique signatures and signatures affiliating them to Eukarya.  Ref: 12107592 J Mol Evol, 2002
- Light is known to stimulate the expression of two unlinked genes for carotenoid synthesis,  **carB**  and carC, through a mechanism in which the regulatory genes  **carA** , carQ and carR take part.  Ref: 7968516 Mol Microbiol, 1993
- The carbamoyl phosphate synthase CPS enzyme in prokaryotes is a heterodimer, encoded by genes commonly called  **carA**  and  **carB** .  Ref: 7773412 Microbiology, 1995
- In Pseudomonas aeruginosa,  **carA**  and  **carB**  are also co transcribed, but are separated by 682 bp.  Ref: 7773412 Microbiology, 1995
- In the bacterium Myxococcus xanthus, several genes for carotenoid synthesis lie together at the  **carA**   **carB**  chromosomal locus and are co ordinately activated by blue light.  Ref: 7588751 Eur J Biochem, 1995
- Transposon Tn5 mutagenesis was used to localize the  **carA**  and  **carB**  genes on the cloned R. meliloti DNA.  Ref: 2846756 J Gen Microbiol, 1988
- Group 2 taxa comprising Halobacteriales, Thermoplasmales, Methanococcales, Methanomicrobiales, Archaeoglobales harbor CPS genes whose encoded  **CarB**  and  **CarA**  subunit proteins are ostensibly bacterial in origin.  Ref: 12107592 J Mol Evol, 2002
- Deletion and transposition analysis determined the locations of  **carA** , encoding the small subunit, and  **carB** , encoding the large subunit, on the chromosomal insert.  Ref: 2153657 J Bacteriol, 1990
- **carA**  1125 bp and  **carB**  3237 bp are similar in size and sequence to other prokaryotic CPS genes, however they are separated by an intervening sequence of 3290 bp which has no similarity to the intervening sequence between other CPS genes.  Ref: 7773412 Microbiology, 1995
- Clones complementing defects in argA, argB, argE, argG, argIF,  **carA** , and  **carB**  were isolated.  Ref: 2493452 J Bacteriol, 1989
- Several neisserial repetitive sequences were identified within the 9 kb sequenced, as well as novel 120 and 150 bp repeats designated RS6 and RS7, respectively which were found within the intervening sequence between  **carA**  and  **carB** .  Ref: 7773412 Microbiology, 1995
- Genetic complementation between the plasmids and mutants demonstrated a single complementation group for  **carA** , but two overlapping complementation groups for  **carB** .  Ref: 2846756 J Gen Microbiol, 1988
- Restriction analysis of these plasmids indicated that complementation involved two unlinked regions of the R. meliloti chromosome,  **carA**  and  **carB** .  Ref: 2846756 J Gen Microbiol, 1988
- The results also show that while  **carA**  and  **carB**  are contiguous in P. stutzeri, as is the case in other bacteria, they are surprisingly separated by an open reading frame ORF of 216 amino acids in P. aeruginosa.  Ref: 8169201 J Bacteriol, 1994
- A construct was made in which the intergenic region between the contiguous  **carA**  and  **carB**  genes was deleted and the sequences encoding the carbamyl phosphate synthetase subunits were fused in frame.  Ref: 9242657 J Biol Chem, 1997
- furthermore, putative transcription terminators are found downstream of both  **carA**  and  **carB** .  Ref: 7773412 Microbiology, 1995
- A similar large, variably sized intervening sequence was also found between the  **carA**  and  **carB**  genes of 12 isolates of N. meningitidis and 18 commensal Neisseria isolates comprising nine species.  Ref: 7773412 Microbiology, 1995
- The intervening sequence was found to vary in size, from approximately 2.2 to 3.7 kb, although the  **carA**  and  **carB**  genes themselves did not vary in size in isolates with functional CPS enzyme.  Ref: 7773412 Microbiology, 1995
- These genes reside on a DNA segment of about 70 kb and are part of a Cb biosynthetic gene cluster that is flanked by two Cb resistance genes,  **carA**  and  **carB** .  Ref: 2628170 Gene, 1989
- A 12 kb DNA stretch from wild type M. xanthus has been sequenced that includes the entire  **carA**   **carB**  gene cluster.  Ref: 7588751 Eur J Biochem, 1995
- We have introduced Tn5 at various sites between  **carA**  and  **carB** .  Ref: 7968516 Mol Microbiol, 1993
- Cocomplementation experiments established gene linkage between  **carA**  and  **carB** .  Ref: 2493452 J Bacteriol, 1989
- that is, they are intermixed with bacterial homologues on a phylogeny of concatenated  **CarA**  and  **CarB**  sequences and are not distinguishable from bacterial sequences after searching for domain specific amino acid residue positions.  Ref: 12107592 J Mol Evol, 2002
- The carAB operon from E. coli hybridized with the gonococcal clones that carried  **carA**  or  **carB**  genes under conditions of high stringency, detecting 80% or greater similarity and showing that the nucleotide sequence of the carbamoylphosphate synthetase genes is very similar in these two organisms.  Ref: 2493452 J Bacteriol, 1989
- The cloned R. meliloti genes hybridize to the corresponding E. coli  **carA**  and  **carB**  genes which encode the two subunits of carbamoylphosphate synthetase.  Ref: 2846756 J Gen Microbiol, 1988
